# Letter from a Distinguished Medical Gentleman

**Published:** 1871-05

**Authors:** 


### LETTER FROM A DISTINGUISHED MEDICAL GENTLEMAN.

We invite attention to a letter in another part of our Journal, with the above caption. We desire to express our thanks to him, as well as others who have communicated to us, almost in the same language, the sentiments and advice he gives.

The effort has been made to prejudice the minds of the uninformed that the controversy to which our correspondent alludes had its inception in "personal matters," as a means of diverting attention from the true issues involved. That such "clap-trap" assertions have had its influence, we are ready to admit—hence the advice of our correspondent is the more important at this juncture, to the professional men at Atlanta, who have been misrepresented in this particular. It will therefore be the policy of this Journal, in the future, as in the past, "never" to "suffer personalities to be dragged in as the means of diverting the minds of the profession from the main issues involved," knowing that personalities injuriously affect the parties resorting to their use, and because the controversy is not a personal one, but, as correctly stated by our friend, "between the profession and the Atlanta Medical College." We desire no higher vindication, *personally*, than the full triumph of the truth, and that of our brethren all over the State who will, for the sake of principle, alone, maintain, not us or our opinions, but the honor and integrity of the profession for which we have labored, regardless of the indifference of friends or the enmity of our foes.

